# Oncogenic and drug-sensitive *RET* mutations in human epithelial ovarian cancer

**DOI:** 10.1186/s13046-020-01557-3

**Published:** 2020-03-23

**Authors:** Luyao Guan, Zhang Li, Feifei Xie, Yuzhi Pang, Chenyun Zhang, Haosha Tang, Hao Zhang, Chun Chen, Yaying Zhan, Ting Zhao, Hongyuan Jiang, Xiaona Jia, Yuexiang Wang, Yuan Lu

**Affiliations:** 1 Department of Gynecology, Obstetrics and Gynecology Hospital, Fudan University Shanghai, 419 Fangxie Rd, Shanghai, 200011 People’s Republic of China; 2 Key Laboratory of Tissue Microenvironment and Tumor, SINH - Changzheng Hospital Joint Center for Translational Medicine, Institutes for Translational Medicine (CAS-SMMU), Shanghai Institute of Nutrition and Health, University of Chinese Academy of Sciences, Chinese Academy of Sciences, 320 Yueyang Rd, Shanghai, 200031 People’s Republic of China; 3 Department of Pathology, Obstetrics and Gynecology Hospital, Fudan University Shanghai, Shanghai, People’s Republic of China

**Keywords:** RET, Ovarian cancer, Vandetanib, Targeted therapy

## Abstract

**Background:**

Epithelial ovarian cancer (EOC) is a highly lethal malignancy. Improvement in genetic characterization of EOC patients is required to propose new potential targets, since surgical resection coupled to chemotherapy, presents several limits such as cancer recurrence and drug resistance. Targeted therapies have more efficacy and less toxicity than standard treatments. One of the most relevant cancer-specific actionable targets are protein tyrosine kinases (PTKs) whose role in EOC need to be better investigated.

**Methods:**

EOC genomic datasets are retrieved and analyzed. The biological and clinical significance of *RET* genomic aberrations in ovarian cancer context are investigated by a series of in vitro and in vivo experiments.

**Results:**

Epithelial ovarian cancer sequencing projects identify recurrent genomic *RET* missense mutations in 1.98% of patients, ranking as the top-five hit among the 100 receptor tyrosine kinases-encoding genes. RET mutants R693H and A750T show oncogenic transformation properties in NIH3T3 cells. Introduction of the RET mutants into human EOC cells increases RET signaling, cell viability, anchorage-independent cell growth and tumor xenograft growth in nude mice, demonstrating that they are activating mutations. RET mutants significantly enhance the activation of RET and its downstream MAPK and AKT signaling pathway in ovarian cancer cells. Vandetanib, a clinical approved RET inhibitor, inhibits the cell viability and decreases the activation of RET-MAPK signaling pathways in EOC cells expressing oncogenic RET mutants.

**Conclusions:**

The discovery of RET pathogenic variants in the EOC patients, suggests a previously underestimated role for RET in EOC tumorigenesis. The identification of the gain-of-function *RET* mutations in EOC highlights the potential use of RET in targeted therapy to treat ovarian cancer patients.

## Background

Ovarian cancer remains the most deadly gynecological malignancy due to the advanced stage at which patients are diagnosed. The number of new cases in 2019 is estimated to be 22,530, and the estimated number of deaths is approximately 13,980 globally [1]. Cytoreductive surgery combined with platinum-based chemotherapy has been the standard of care for advanced ovarian cancer for the last 35 years [2]. Although patients are sensitive to platinum initially, they develop platinum resistance after multiple relapses, and the effectiveness of second-line chemotherapy is limited [3]. In addition, increasing evidence has shown that ovarian cancer should not be regarded as a single entity, as different subtypes have distinct clinical, histological, and molecular features [4]. Compared with empirical therapy, molecularly targeted therapy might be a more effective and less toxic therapy for ovarian cancer patients.

PARP inhibitors (PARPi) and antiangiogenic agents are the two main kinds of molecularly targeted drugs introduced as frontline therapy for ovarian cancer [4]. PARPi demonstrated significant promotion of progression-free survival (PFS) and the latest reported overall survival (OS) period when used as a maintenance therapy in recurrent ovarian cancer patients; the benefits are mainly shown in platinum-sensitive patients or patients with *BRCA* mutations [5], and patients without HRD (homologous recombination deficiency) may not benefit from PARPi according to the “synthetic lethal” theory. Antiangiogenic agents mainly include monoclonal antibodies such as bevacizumab targeting vascular endothelial growth factor (VEGF) and tyrosine kinase inhibitors (TKIs) targeting VEGF receptor (VEGFR). Antiangiogenic therapies have been integrated into the treatment of ovarian cancer patients per the recommended guidelines, but the OS benefits need to be identified considering the cost-effectiveness and toxicity. There have been no licensed targeted agents for ovarian cancer since bevacizumab was approved in 2014 [6], which encouraged us to explore whether there are other targets for the treatment of ovarian cancer patients.

Protein tyrosine kinases (PTKs) genes are a major kind of oncogene divided into transmembrane receptor tyrosine kinases (RTKs) and cytoplasmic nonreceptor tyrosine kinases (NRTKs) genes. PTKs genes are involved in survival, proliferation, invasion, and angiogenesis in many cancers, making them potential therapeutic targets in cancer treatments. With the deeper understanding of kinases and the faster development of pharmaceuticals, there were 19 kinase inhibitors approved in 4 years (from 2011 to 2015, [7]). Taking gefitinib as an example, it was licensed to treat non-small-cell lung cancer (NSCLC) patients with epidermal growth factor receptor (EGFR) mutations and yielded a significant PFS benefit (10.4 versus 5.5 months) compared with the chemotherapy group [8]. These encouraging facts led us to explore the oncogenic role of PTKs in ovarian cancer.

**Fig. 1:**
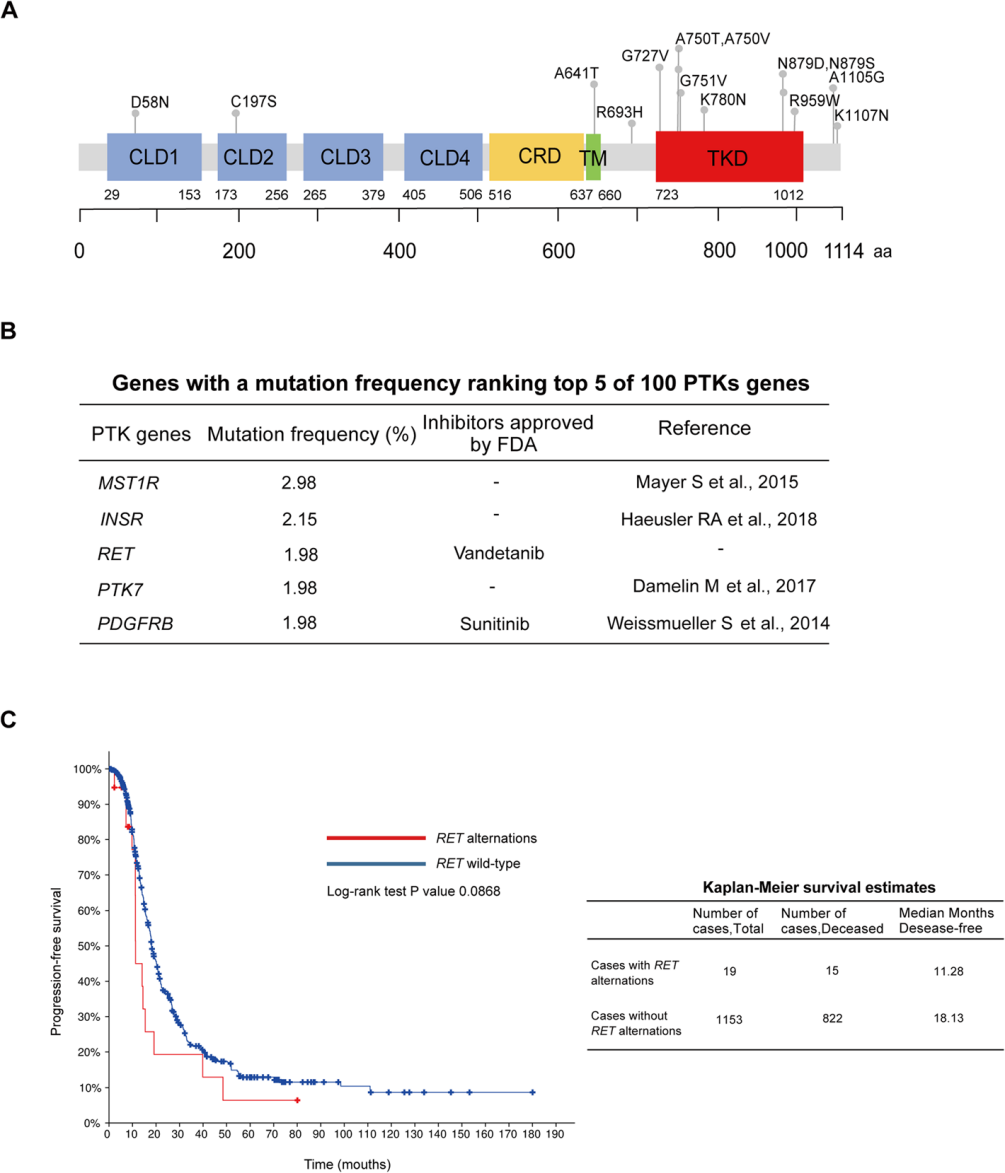
*RET* alterations in ovarian cancer. **a** Missense mutations in the CDS (coding sequence) of *RET* from the TCGA, COSMIC, ICGC and CCLE databases are marked over the affected protein domains. CLD, cadherin-like domain; CRD, cysteine-rich domain; TM, transmembrane domain; TKD, tyrosine kinase domain. **b** Genes with a mutation frequency ranking in the top 5 of 100 PTKs in ovarian cancer are listed, and the mutation frequency, FDA approved inhibitors and representative reference are also shown. **c** Kaplan-Meier plot of the progression-free survival of ovarian cancer patients based on *RET* alterations from the TCGA database

RET (rearranged during transfection) is a single transmembrane RTK that consists of an extracellular domain containing four cadherin-like domains and a cysteine-rich domain, a transmembrane domain, and an intracellular kinase domain [9] (Fig. 1a). As a typical RTK, mutation, rearrangement, and aberrant expression of the *RET* gene induces the autophosphorylation of RET, and then phosphorylated RET phosphorylates downstream signaling pathways to drive various cancers, such as hereditary and sporadic medullary thyroid carcinoma (MTC) [10, 11], papillary thyroid cancers [12], and NSCLC [13]. Vandetanib, a TKI (tyrosine kinase inhibitors) with inhibitory activity against RET, has been approved for the treatment of patients with locally advanced and metastatic MTC whose pathogenesis mainly comes from *RET* mutations [14]. A phase III clinical trial showed that MTC patients with *RET* mutations benefited more from vandetanib [15], which suggested vandetanib might serve as a therapeutic choice for patients with *RET* alterations. We studied the oncogenic functions of *RET* mutations and tested the therapeutic effects of vandetanib in ovarian cancer.

As epithelial ovarian cancer (EOC) accounts for over 95% of ovarian cancer [16], we focused on EOC in this study. First, we collected and analyzed data on *RET* mutations in EOC from genomic databases and found the oncogenic effects of *RET* mutations on promoting cell viability and colony formation of ovarian cancer cells in vitro and accelerating tumor xenograft growth of ovarian cancer in vivo. Next, we demonstrated that the kinase inhibitor vandetanib could effectively inhibit the viability of ovarian cancer cells with *RET* mutations. The RET-MAPK signaling pathway might be the main mechanism by which RET mutants promote the development of ovarian cancer. Our study identified the oncogenic role of *RET* mutations in ovarian cancer and provided a potential treatment option for ovarian cancer patients with *RET* mutations.

## Methods

### Identification of potential active mutations

To identify *RET* mutations in ovarian cancer, we searched widely used genomic databases, including TCGA (The Cancer Genome Atlas), COSMIC (Catalogue of Somatic Mutations in Cancer), ICGC (International Cancer Genome Consortium), and CCLE (Broad Institute Cancer Cell Line Encyclopedia). To filter potential active mutations, we set two criteria: 1) the mutations predicted to be “possibly damaging” or “probably damaging” in PolyPhen-2 or predicted to be “damaging” in SIFT (Sorting Intolerant from Tolerant) were included, and 2) the amino acid substitutions presented in the 1000 Genomes Project were excluded.

### Construction of mutant vectors and packaging lentivirus

The PCDH3.1-RET51-WT (wild-type) plasmid was a generous gift from Professor Lois M. Mulligan. This plasmid was digested with NOT1 (#R3189S, New England Biolabs) and NheI (#R3131S, New England Biolabs) restriction endonucleases to obtain the coding sequences (CDS) of *RET*. The pCDH-CMV-MCS-EF1-Neo plasmid (#CD514B-1, System biosciences) was used as the empty vector (EV) and was treated in the same way, and then the products were ligated using T4 DNA Ligase (M0202S, NEB) to gain pCDH-RET51-WT plasmid (WT). RET mutants were constructed by site-directed mutagenesis of the pCDH-RET51-WT plasmid using the QuikChange Lightning Site-Directed Mutagenesis Kit (#210518, Agilent). The constructs with *RET* mutations were confirmed by sequencing. The lentivirus particles containing EV, WT, RET mutants were generated as described [17].

### Cell culture and transfection

HEK293T cells (#ACS-4500), NIH3T3 cells (#CRL-1658) and pancreatic ductal epithelia cell line MiaPaCa-2 (#CRL-1420) were purchased from ATCC. The EOC cell line A2780 and SKOV3 was obtained from the Cell Bank of the Chinese Academy of Sciences (Shanghai, China) and OVK18 was purchased from RIKEN (RCB1903). All cells were validated by short-tandem-repeat (STR) DNA fingerprinting. HEK293T cells, NIH3T3 cells, A2780 cells and SKOV3 cells were supplemented with RPMI 1640 (#11875093, Thermo Fisher Scientific). MiaPaCa-2 were supplemented with DMEM/F-12 (#11330057, Thermo Fisher Scientific). OVK18 cells were cultured with MEM (#11095080, Thermo Fisher Scientific). All above media contain 10% fetal bovine serum (#10099141, Thermo Fisher Scientific) and streptomycin/penicillin and maintained in a 5% CO2 humidified atmosphere at 37 °C. All cell lines were routinely tested for microbial contamination (including mycoplasma). None of the cell lines in this study appears in the misidentified cell line list kept by the ICLAC.

For transient transfection, 1 μg constructed plasmids were transfected into HEK293T cells using Lipofectamine 2000 (#11668019, Thermo Fisher Scientific) according to the manufacturer’s instructions. For stable transfection, cells were transduced with RET mutants when cell confluence reached 50% and were selected with 2 μg/ml puromycin for 4 days before use.

### Western blot analysis

Cells were lysed with lysis buffer (1% NP-40, 50 mM Tris-HCl pH 8.0, 100 mM sodium fluoride, 30 mM sodium pyrophosphate, 2 mM sodium molybdate, 5 mM ethylenediaminetetraacetic acid and 2 mM sodium orthovanadate) containing protease inhibitors (leupeptin, aprotinin, and phenylmethylsulfonyl fluoride). Lysates were quantified using the Quick Start Bradford 1× Dye Reagent (#5000205, Bio-Rad). Equal amounts of protein were separated on 10% SDS-polyacrylamide gels and then transferred to nitrocellulose membranes. The membranes were incubated with primary antibodies at 4 °C overnight and blocked with HRP-conjugated secondary antibody at room temperature for 1 h. The signals were detected using a chemiluminescence kit (Immobilon Western, Millipore Corporation, MA). Quantification of band intensity was analyzed by Image Quant TL 8.1 software (GE Healthcare).

### Antibodies and chemicals

The primary antibodies used in this study included anti-phospho-RET (Tyr905, #3221), anti-RET (#14556), anti-phospho-ERK (Thr202/Tyr204, #9101S), anti-ERK (#9102), anti-phospho-AKT (Ser473, #9271) and anti-AKT (#9272). They were purchased from Cell Signaling Technology. Anti-GAPDH (# G8795) was purchased from sigma.

Vandetanib was purchased from Selleck Chemicals and diluted with DMSO to 6 concentrations: 0, 500, 750, 1000, 2000, and 5000 nM. For vandetanib inhibition test, A2780 cells transfected with RET mutants were cultured with different concentrations of vandetanib for 72 h. For western blotting, A2780 cells transfected with EV, WT or RET mutants were treated with 500 nM vandetanib for 4 h before harvesting.

### Cell Titer-Glo (CTG) assay

A2780 cells (2000 cells per well) transfected with EV, WT or RET mutants were seeded in 96-well plates in triplicate. Cell viability was determined using CTG (#G7572, Promega) according to the manufacturer’s instructions. The relative cell viability was calculated as the cell viability at 96 h relative to that at 24 h. The inhibition effect of vandetanib was calculated as the cell viability of cells treated with concentrations of 500, 750, 1000, 2000, and 5000 nM relative to that of cells treated with 0 nM at 96 h. The experiments were repeated three times.

### Soft agar assay and plate clone formation assay

To examine anchorage-independent growth, NIH3T3 cells (2 × 10^5^ cells per well) transfected with EV, WT or RET mutants were suspended in 0.35% agar (#214220, BD Difco) containing RPMI 1640 medium with 10% fetal bovine serum and penicillin/streptomycin and seeded on 0.7% agar with the same complete media in 6-well plates. Cells were allowed to grow for 5 weeks and stained with methyl thiazol tetrazolium (#M5655, Sigma-Aldrich) for 3 h. The number of colonies was counted manually. This assay was performed in triplicate.

For plate clone formation assay, A2780 cells (500 cells per well) transfected with EV, WT or RET mutants were seeded in 6-well plates in triplicate for 14 days. Cells were fixed with methanol for 15 min and stained with crystal violet for 30 min. The number of colonies was counted manually. This experiment was performed three times.

### Xenograft assays

The xenograft experiment was approved by the Animal Ethics Committee of Fudan University. Five-week-old female nude mice (BALB/c nude, SLAC, Shanghai, China) were randomly divided into 4 groups (4 mice per group) and inoculated subcutaneously into the left flank with A2780 cells (3 × 10^6^ cells per mice) transfected with EV, WT or RET mutants in 50 μl PBS and an equal volume of Matrigel (#356234, Corning). All mice were sacrificed 3 weeks later, and the tumors were removed to measure volume and weight. Tumor volume was calculated using the formula: Tumor volume = length×width^2^/2. This experiment was repeated three times.

### Statistical analysis

Statistical analysis and graph generation were performed using GraphPad Prism 7.0. Unpaired two-tailed Student’s t-test was used to evaluate the significance of differences between two groups. A *P*-value < 0.05 was considered statistically significant.

## Results

### Recurrent *RET* mutations identified in ovarian cancer patients

To search for PTKs-encoding gene mutations with oncogenic potential in ovarian cancer, we collected and analyzed genome sequencing data from the TCGA (*n* = 605) and found that the genes with a mutation frequency ranking in the top 5 of 100 PTK genes (Fig. 1a; Additional file 1: Supplementary Figure 1 and Additional file 2: Supplementary Table 1) in epithelial ovarian cancer were *MST1R, INSR, RET, PDGFRB*, and *PTK7,*among which *RET* (reference transcript, NM_02097; reference protein, NP_066124) gene mutations had not been studied in ovarian cancer patients and had approved inhibitors (Fig. 1b; Additional file 2: Supplementary Table 1). Endogenous RET is expressed in epithelial ovary cancer at both RNA and protein levels (Additional file 1: Supplementary Figure 2A-2B).

Patients with *RET* gene mutations were identified in 12 out of 605 (1.98%) ovarian cancer patients (Additional file 2: Supplementary Table 2), and those with *RET* alterations had shorter progression-free survival than those without *RET* alterations (11.27 versus 18.13 months; Fig. 1c), which indicated that *RET* alterations play a role in the progression of ovarian cancer. To obtain a comprehensive view of *RET* mutations in ovarian cancer, we also collected *RET* mutations from other sequencing databases, including COSMIC, ICGC and CCLE. There were 22 missense mutations in total. To select potential activating *RET* mutations among these mutations, we used Polyphen2 [18] and SIFT [19], two commonly used prediction algorithms, to predict whether specific amino acid substitutions of RET would affect protein function based on the sequence homology and structure of protein. Ultimately, 14 missense mutations were further studied, as other substitutions were either predicted to be benign according to the scores of PolyPhen-2 and SIFT (R79Q, R114H, R205S, G248S, A342G, T636M, T1085A, and A680T) or presented in the 1000 Genomes Project (A680T) (Additional file 2: Supplementary Table 3).

### Oncogenic transformation properties of *RET* mutations

**Fig. 2:**
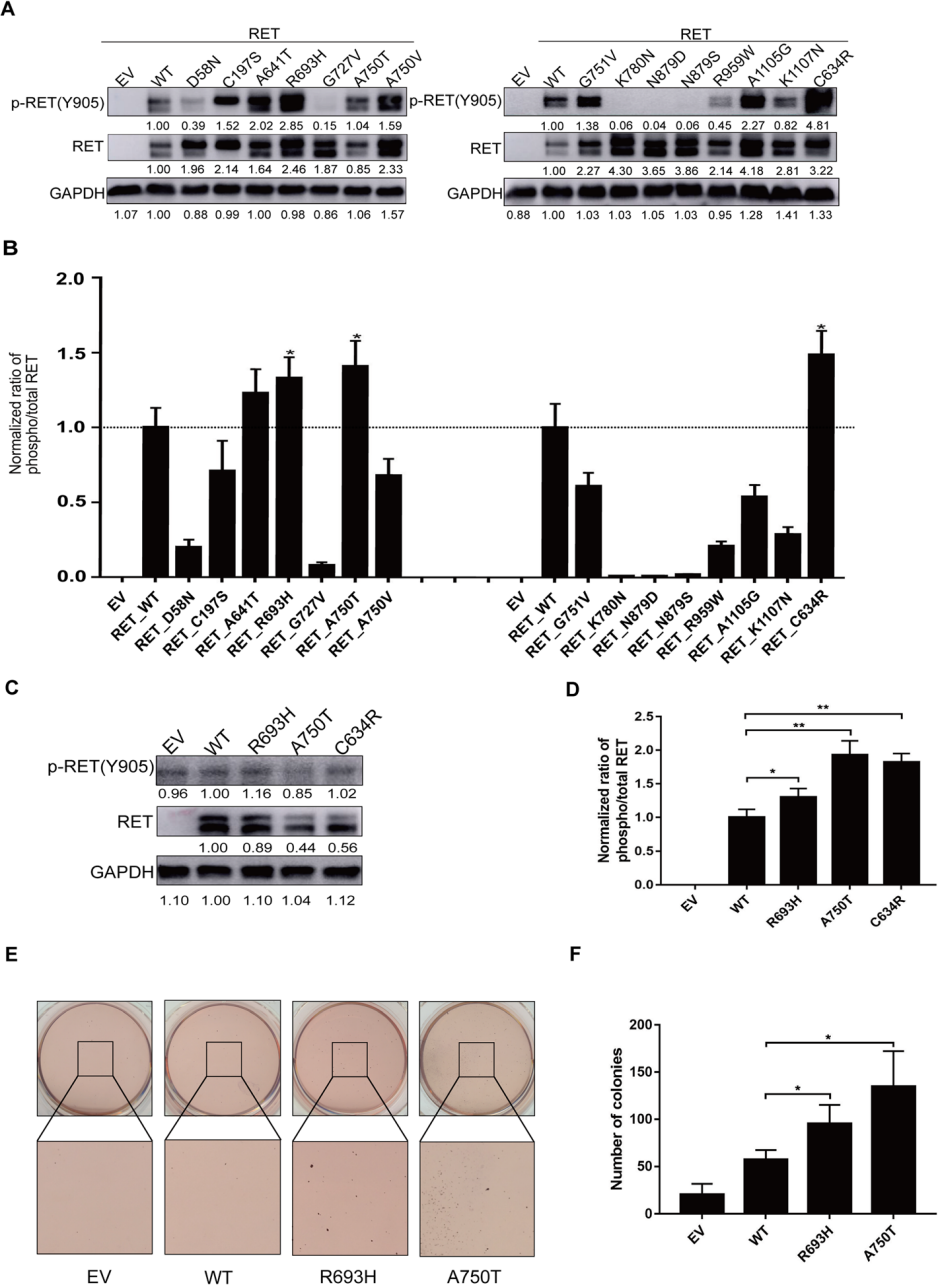
Oncogenic transformation properties of *RET* mutations. **a** HEK293T cells were transfected transiently with empty vector (EV), RET wild-type (WT) or RET mutants (14 potential active mutations and C634R mutant as the positive control). The lysates were analyzed by western blotting with anti-phospho RET (Y905) and anti-RET antibodies. **b** Bar graphs demonstrated the quantification of western blotting bands in (**a**), normalized to WT control. **c** NIH3T3 cells were transfected stably with empty vector (EV), RET wild-type (WT) or RET mutants (R693H and A750T) viruses. The lysates were analyzed by western blotting with anti-phospho RET (Y905) and anti-RET antibodies. **d** Bar graphs showed the quantification of western blotting bands in (**c**), normalized to WT control. **e** and **f** RET mutants promote the anchorage-independent growth of NIH3T3 cells. NIH3T3 cells stably transfected with EV, WT, or RET mutants were seeded in soft agar in triplicate for 5 weeks and stained with methyl thiazol tetrazolium (MTT), representative plates (**f**) and the number of colonies (**f**) are shown. All of the above results represent three repeated experiments *, *P* < 0.05 compared to WT

To study the oncogenic potentials of the 14 mutations, we transiently transfected plasmids expressing these mutations into HEK293T cells and detected their effects on the phosphorylation of RET. C634R was used as the positive control of the oncogenic *RET* mutation [20, 21]. R693H, A750T and C634R mutants significantly increased the ratio of phosphorylated RET to total RET compared with that of cells transfected with wild type RET (WT) or empty vector (EV) (Fig. 2a and b), which indicated that these two mutations were able to activate RET*.* A641T seemed not an active mutation because it is not able to activate RET in HEK293T cells or NIH3T3 cells (Additional file 1: Supplementary Figure 3A-3C).

To further identify the effects of R693H and A750T on anchorage-independent growth, we used the NIH3T3 mouse embryo fibroblast cell line as a model that is commonly used to evaluate the transforming ability of oncogenes [22]. Additionally, NIH3T3 cells do not express endogenous RET [21] (Additional file 1: Supplementary Figure 2B). We constructed lentiviral vectors encoding RET mutants and transduced them into NIH3T3 cells. R693H and A750T significantly increased the phosphorylation of RET in the Western blot, demonstrating that RET was activated in NIH3T3 cells (Fig. 2c and d). The colony formation assay showed that NIH3T3 cells transduced with EV and WT vectors formed few colonies in soft agar, while R693H and A750T vectors significantly increased the number of colonies (Fig. 2e and f), which demonstrated that R693H and A750T promote anchorage-independent growth and that both of them are gain-of-function mutations.

### RET mutations activate RET signals and promote ovarian cancer growth in vitro and in vivo

**Fig. 3:**
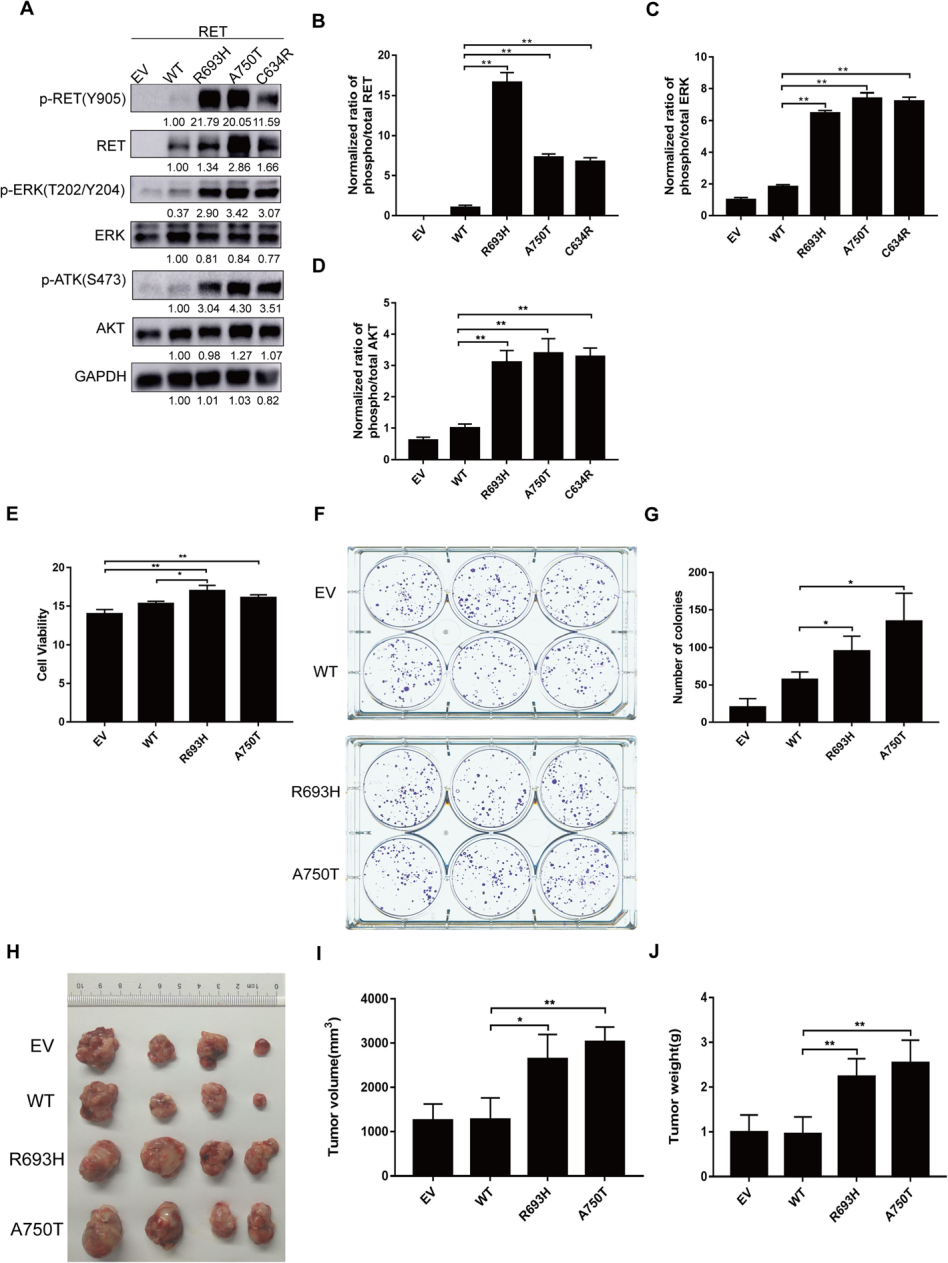
Oncogenic potential of RET mutants in vitro and in vivo. **a** Lysates from epithelial ovarian cancer cell (EOC) A2780 transduced with EV or WT or mutants were analyzed by immunoblot with anti-phospho RET (Y905), anti-RET antibody, anti-phospho ERK (T202/204), anti-ERK antibody, anti-phospho AKT (S473), and anti-AKT antibody (**b**-**d**) Bar graphs showing quantification of western blot bands in (**a**), normalized to WT control. **e** R693H and A750T mutants increase the cell viability of A2780 cells. A2780 cells stably expressing EV, RET WT or RET mutants grew on 96-well plates in triplicate for 72 h, and the viability was measured by CTG assay. **f** and **g** A2780 cells transduced with EV, RET WT or mutants were seeded in 6-well plates (500 cells per well) in triplicate for 2 weeks and stained with MTT. Representative plates (**f**) and number of colonies are presented (**g**). **h**–**j** RET mutants promote the growth of ovarian cancer xenografts in nude mice. Representative pictures (**h**), tumor volumes (**i**) and tumor weights (**j**) are shown. *, *P* < 0.05 compared to WT. **, *P* < 0.01 compared to WT

To further evaluate the oncogenic functions of R693H and A750T in ovarian cancers, we transduced lentiviral vectors expressing these mutants or WT or C634R mutant (positive control [23]) into an EOC cell line A2780. The A2780 cell line is a widely used, low-RET-expressing cell line in the CCLE. Western blot analysis showed that the R693H or A750T mutant significantly elevated the phosphorylation of RET. As the MAPK and AKT signaling pathways are the main downstream signal transduction pathway of RET, we detected the ratio of phosphorylated ERK to total ERK and phosphorylated AKT to total AKT to assess the activation of downstream signal pathways and found that cells expressing the R693H or A750T mutant had higher ERK phosphorylation than cells expressing WT (Fig. 3a-c), which confirmed that both mutations promote the activation of the MAPK and AKT signaling pathways.

A CTG assay was performed to investigate the role of R693H and A750T mutations in promoting cell viability. We found that viability was higher in the R693H or A750T mutant-expressing A2780 cells than in the WT-expressing cells (Fig. 3d). A plate colony formation assay showed similar results: the number of colonies was higher in A2780 cells transduced with R693H or A750T mutant than in cells transduced with WT (Fig. 3e-f). Next, we investigated the oncogenic role of *RET* mutations in the growth of tumor xenografts in nude mice and found that WT and EV barely increased the growth of A2780 cells. In contrast, A2780 cells expressing the R693H or A750T mutant significantly increased the volume and weight of tumors compared with those of WT (Fig. 3g-i).

In conclusion, R693H and A750T mutants of *RET* enhance the signal transduction of RET, the cell viability and colony formation of cells, and the growth of tumor xenografts of ovarian cancer.

### Vandetanib inhibits the viability and reduces the cell signaling of ovarian cancer cells expressing R693H or A750T mutants

**Fig. 4:**
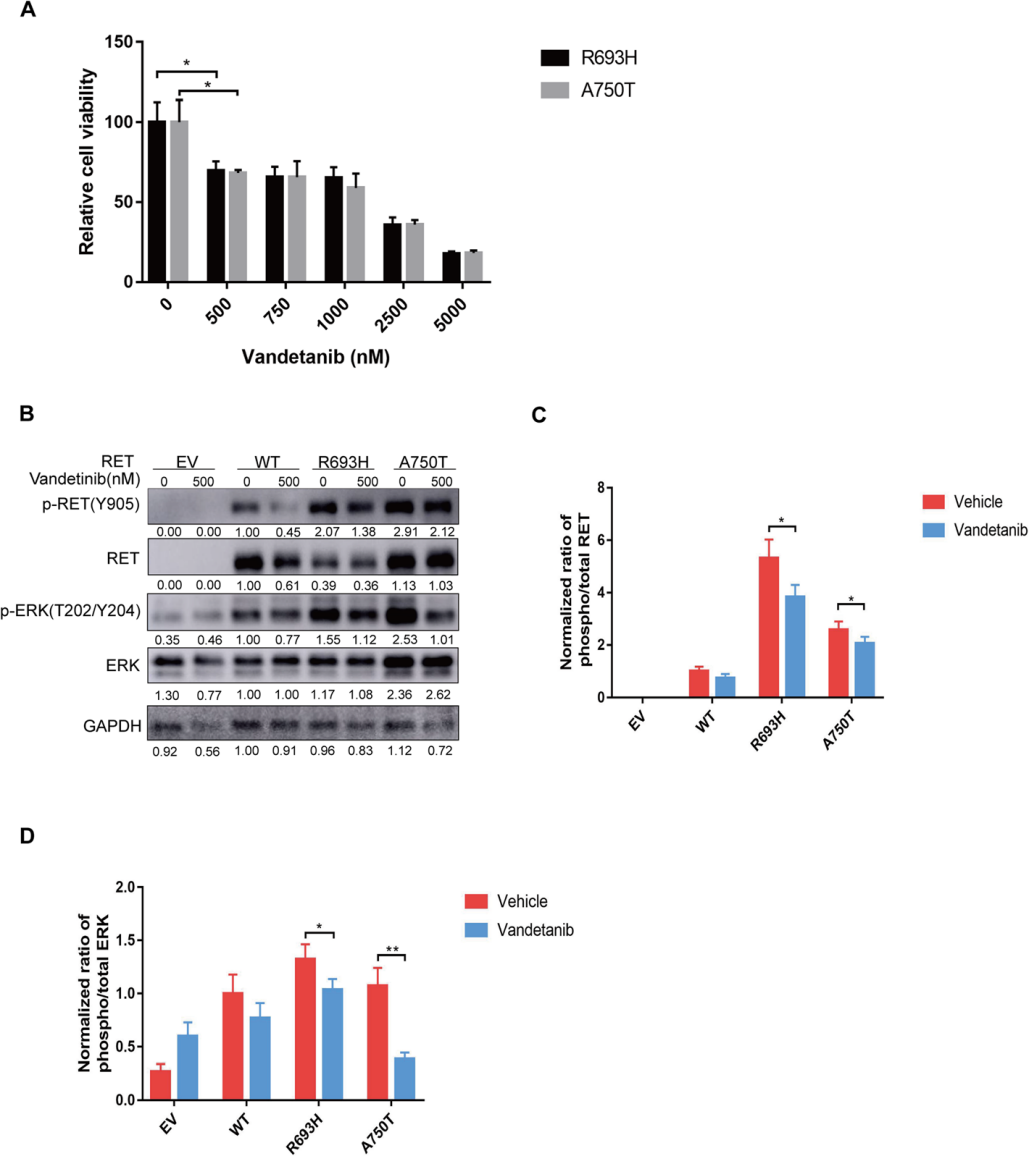
Vandetanib inhibits the viability and cell signaling of RET in EOC cells expressing RET mutants. **a** Drug sensitivity of RET mutants in the CTG assay. A2780 cells transduced with RET R693H or A750T were seeded in 96-well plates in triplicate and treated with 0, 500, 750, 1000, 2500, or 5000 nM vandetanib at 24 h and 72 h after plating. The inhibition effects of vandetanib are shown in the bar graph and normalized to the WT control. **b** Vandetanib strongly inhibits the phosphorylation of RET and ERK in A2780 cells with RET mutations. A2780 cells expressing either RET R693H or A750T mutant were treated with 500 nM vandetanib for 4 h before harvesting, and the western blotting results are shown in (**b**). **c** and **d** Bar graphs showing quantification of western blotting bands in (**b**), normalized to WT control. The results represent three repeated experiments *, *P* < 0.05 compared to WT. **, *P* < 0.01 compared to WT

Vandetanib is an inhibitor with strong activity against RET, EGFR, and VEGFR, which demonstrated more benefit in MTC patients with *RET* mutations than in patients without *RET* mutations. To investigate whether vandetanib has the potential to be applied in ovarian cancer patients with *RET* mutations, we tested the inhibitory effects of vandetanib on A2780 cells expressing the R693H or A750T mutant. The results showed that vandetanib dramatically decreases cell viability at a concentration of 500 nM and revealed a dose-dependent relationship between vandetanib and viability (Fig. 4a). To determine the molecular mechanisms of the inhibitory effects, western blotting was performed and demonstrated that vandetanib reduces the phosphorylation of RET and ERK in A2780 cells expressing the R693H or A750T mutant (Fig. 4b-d), which was consistent with the conclusion that the R693H or A750T mutant enhances RET signal transduction. We inferred that vandetanib has the potential to treat EOC patients with RET gain-of-functional mutations.

## Discussion

Debulking surgery combined with platinum-based chemotherapy as an empirical treatment for advanced ovarian cancer has been used for the last 35 years, while the 5-year survival of late-stage ovarian cancer is still < 30% [1]. Both the lack of effective treatments after the development of platinum resistance and the heterogeneity of ovarian cancer pushed us to explore the potential of targeted therapy for ovarian cancer. Additionally, targeted therapy has the advantage of improving therapeutic effects and reducing therapeutic toxicity [23]. PARPi and bevacizumab are two primary molecularly targeted agents in ovarian cancer. Both of these agents demonstrated PFS benefit, and PARPi also showed OS benefit in platinum-sensitive recurrent ovarian cancer patients [5]; however, they both lack definite biomarkers to predict therapeutic outcome, which suggests that neither could represent virtually personalized therapy [24].

PTKs are the main targets in the targeted treatment of cancer. The first approved targeted agent was trastuzumab to treat HER2-positive breast cancer patients in 1998 [25]. Since then, an increasing number of PTKs have been recognized as therapeutic targets, such as the “miracle” drug imatinib which targets BCR-ABL. Its emergence improved the complete cytogenetic response of Philadelphia chromosome-positive chronic myelogenous leukemia by almost 50% [26]. The remarkable effects and the emergence of PTKs inhibitors provides us with an opportunity to study whether PTKs could be regarded as potential targets in ovarian cancer.

In our study, we identified the genes with a mutation frequency ranking in the top 5 of 100 PTK genes in EOC, namely, *MST1R, INSR, RET, PDGFRB,* and *PTK7,* among which we studied the overlooked oncogenic role of *RET* in ovarian cancer. Our results showed that RET R693H and A750T mutants could promote the viability, colony formation and activation of the RET-MAPK and RET-AKT signaling pathway in ovarian cancer, and the RET inhibitor vandetanib could significantly decrease cell viability and signal transduction at 500 nM. Our study provides a good starting point for the application of RET inhibitors in EOC patients with *RET* mutations.

The total RET and total ERK protein levels were different among mutants as shown in Figs. 2a and 3a, which may be explained by the different lentiviral infection efficiency (due to different virus titers) among mutants. The phosphorylated protein and total protein were loaded proportionally in the same western blot experiment. Hence the ratio of phosphorylated protein/total protein was used to prove the activation effects of various *RET* mutations.

R693H and A750T are located in the juxtamembrane (JM) region and intracellular kinase domain, respectively. Mutations in the kinase domain were commonly reported in MTC, including the most common *RET* mutation M918T, which was associated with more severe disease phenotypes of MTC [27]. The oncogenic mutations in this domain may induce conformational changes in proteins, which decrease autoinhibition, increase kinase activity and ATP binding, generate a better intermolecular substrate [28, 29], and then cause the autophosphorylation of RET to induce cancer. There are some publications about the polymorphism in the JM region of RET. The G691S RET polymorphism was reported to enhance the response of RET to glial cell line-derived neurotrophic factor (GDNF) and was correlated with the aggressive phenotype of pancreatic cancers [30] and cutaneous malignant melanomas [31]. This substitution is close to the RET R693H mutant we studied, which reflects the importance of variations in this domain in cancer, while the allosteric mechanisms are not yet known. Interestingly, there was a decrease in the level of RET protein after vandetanib treatment, especially in the WT-expressing cells (Fig. 4b). How the vandetanib decreases the expression of total WT RET warrants further investigation.

There are some potential limitations to our study: 1) Compared with other targets, the mutation frequency of PTKs genes, including *RET,* is low, while according to the conception of precision therapy, we believe that patients with *RET* mutations would benefit from the personalized treatment strategy targeting RET. International cooperation and case sharing will facilitate the application of PTKs inhibitors. 2) Most currently used PTKs inhibitors, including vandetanib, are multikinase inhibitors, which might influence the therapeutic specificity and thus weaken the curative efficacy. The development of next-generation selective RET inhibitors such as BLU-667 [32] and LOXO-292 [33] may contribute to the precision therapy of patients with *RET*-mutant ovarian cancer. Taking BLU-667 as an example, it was reported to have 88-fold more potency against RET than VEGFR [34], and it has already demonstrated clinical benefits in *RET*-altered thyroid cancer and lung cancer patients in early stage clinical trials [35, 36] .

It has been only 5 years since the first FDA-approved targeted medicine emerged in ovarian cancer. To date, the effects of PTKs inhibitors in ovarian cancer patients in early-stage clinical trials have been modest compared with the effects of therapies targeting oncogenic drivers in other solid tumors [37]. The explanation might come from the lack of patient selection in the trials. In addition, the heterogeneity creates a more complex landscape of ovarian cancer, so combination therapy with other chemotherapies or targeted drugs might be helpful, and the determination of a precise treatment based on the dynamic characteristics of the genome of ovarian cancer would also be beneficial [38].

The application of next-generation genetic sequencing technology, the unceasing exploration of genetics, the expansion of noninvasive biomarker detection technology [39, 40], and the dramatic development of the pharmaceutical industry will accelerate the development of precision medicine for ovarian cancer and eventually reduce the morbidity and mortality of ovarian cancer patients. We believe that, in the future, therapies targeting RET will prolong survival while not influencing the quality of life of patients with RET-mutant ovarian cancer.

## Conclusions

The discovery of RET pathogenic variants in the EOC patients, suggests a previously underestimated role for RET in EOC tumorigenesis. The identification of the gain-of-function *RET* mutations in EOC highlights the potential use of RET in targeted therapy to treat ovarian cancer patients.

## Supplementary information

**Additional file 1: Supplementary Figure 1.** PTKs-encoding genes. **Supplementary Figure 2.** Endogenous RET expression in human ovarian cancer cells. (A) The RNA-seq data from epithelial ovary carcinoma patients from the TCGA program (TCGA Pan Can Atlas study: *n* = 585; TCGA Provisional study; *n* = 606) were retrieved and analyzed. Endogenous RET is expressed in epithelial ovary carcinoma at RNA level. (B) Lysates from two epithelial ovarian cancer cell lines, SKOV3 and OVK18, were analyzed by western blot with anti-RET antibody and GAPDH antibody. The pancreatic ductal epithelia cell line MiaPaCa-2 and NIH3T3 cell line were positive and negative controls, respectively. **Supplementary Figure 3.** A641T mutant does not activate RET kinase. (A) NIH3T3 cells were transduced stably with EV, RET WT or mutants (A641T and C634R). The lysates were analyzed by western blotting with anti-phospho RET (Y905) and anti-RET antibodies. (B) Bar graphs demonstrated the quantification of western blotting bands in sFigure 3A, normalized to WT control. (C) A641T mutant is not able to increase the cell viability of NIH3T3 cells. NIH3T3 cells stably expressing EV, RET WT, A641T, and C634R (positive control) were cultured on 96-well plates and the viability was measured by CTG assay at day 2.

**Additional file 2: Supplementary Table 1.** Detailed genomic information of 100 PTKs genes and the mutation frequency in 605 EOC patients from TCGA. **Supplementary Table 2.** List of *RET* missense mutations identified in EOC patients from the TCGA. **Supplementary Table 3.** Summary of all *RET* missense mutations identified in EOC patients.

## Data Availability

All data generated or analyzed during this study are included in this published article and its supplementary information file. Further details are available from the corresponding author on reasonable request.

## Abbreviations

PTKs: Protein tyrosine kinases
EOC: Epithelial ovarian cancer
PARPis: Poly ADP-ribose polymerase inhibitors
VEGF: Vascular endothelial growth factor
EGFR: Epidermal growth factor receptor
GDNF: Glial cell line-derived neurotrophic factor
NSCLC: Non-small-cell lung cancer
MTC: Medullary thyroid carcinoma
FDA: Food and Drug Administration
PFS: Progression-free survival
OS: Overall survival
TCGA: The Cancer Genome Atlas
COSMIC: Catalogue of Somatic Mutations in Cancer
ICGC: International Cancer Genome Consortium
CCLE: Broad Institute Cancer Cell Line Encyclopedia
