# Supplementary material for: Oncogenic and drug-sensitive RET mutations in human epithelial ovarian cancer
Source: J Exp Clin Cancer Res. 2020 Mar 23;39:53. doi: 10.1186/s13046-020-01557-3 (PMC7092606; doi:10.1186/s13046-020-01557-3)

Supplementary Figure 1

PTKs-encoding genes

Receptor tyrosine kinases genes (n=60)

AATK ALK AXL CSF1R DDR1 DDR2 EGFR EPHA1 EPHA2 EPHA3 EPHA4 EPHA5 EPHA6 EPHA7 EPHA8 EPHA10 EPHB1 EPHB2 EPHB3 EPHB4 EPHB6 ERBB2 ERBB3 ERBB4 FGFR1 FGFR2 FGFR3 FGFR4 FLT1 FLT3 FLT4 IGF1R INSR INSRR KDR KIT LTK LMTK2 LMTK3 MERTK MET MST1R MUSK NPR1 NPR2 NTRK1 NTRK2 NTRK3 PDGFRA PDGFRB PTK7 RET ROR1 ROR2 RYK STYK1 CTEK TIE1 TYRO3

Non-receptor tyrosine kinases genes (n=40)

ABL1 ABL2 BLK BMX BTK CSK FER FES FGR FRK FYN HCK ILK IRAK4 ITK JAK1 JAK2 JAK3 KSR1 LCK LYN MA3K20 MATK PKDCC PLK4 PTK2 PTK2B PTK6 SRC SRMS SYK TEC TEX14 TNK1 TNK2 TNNI3K TXK TYK2 YES1 ZAP70

Supplementary Figure 2

A

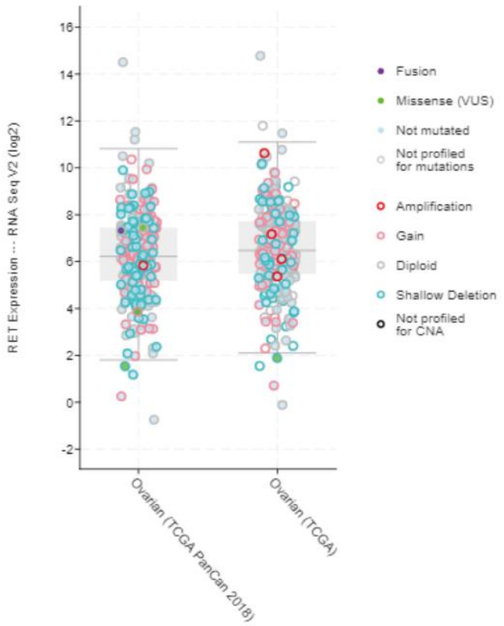

B

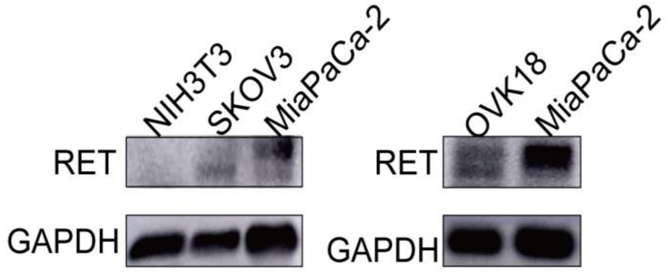

Supplementary Figure 3

A

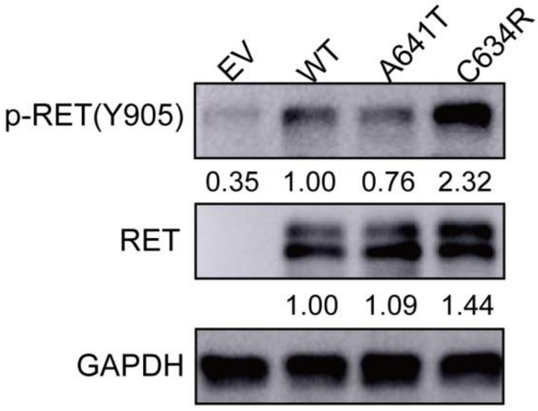

B

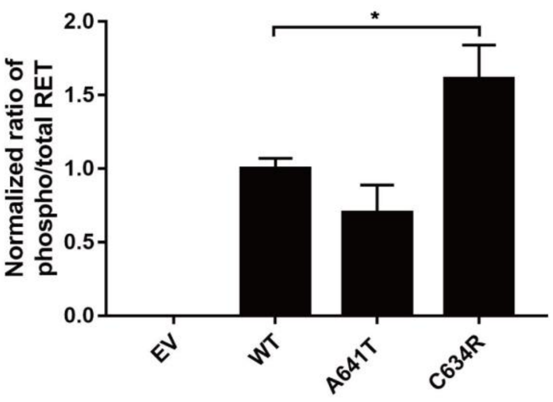

C

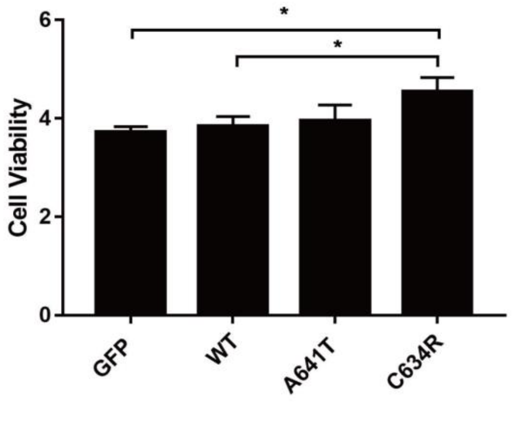

Supplement: Supplementary file 1 — Additional file 1: Supplementary Figure 1. PTKs-encoding genes. Supplementary Figure 2. Endogenous RET expression in human ovarian cancer cells. (A) The RNA-seq data from epithelial ovary carcinoma patients from the TCGA program (TCGA Pan Can Atlas study: n = 585; TCGA Provisional study; n = 606) were retrieved and analyzed. Endogenous RET is expressed in epithelial ovary carcinoma at RNA level. (B) Lysates from two epithelial ovarian cancer cell lines, SKOV3 and OVK18, were analyzed by western blot with anti-RET antibody and GAPDH antibody. The pancreatic ductal epithelia cell line MiaPaCa-2 and NIH3T3 cell line were positive and negative controls, respectively. Supplementary Figure 3. A641T mutant does not activate RET kinase. (A) NIH3T3 cells were transduced stably with EV, RET WT or mutants (A641T and C634R). The lysates were analyzed by western blotting with anti-phospho RET (Y905) and anti-RET antibodies. (B) Bar graphs demonstrated the quantification of western blotting bands in sFigure 3A, normalized to WT control. (C) A641T mutant is not able to increase the cell viability of NIH3T3 cells. NIH3T3 cells stably expressing EV, RET WT, A641T, and C634R (positive control) were cultured on 96-well plates and the viability was measured by CTG assay at day 2. [file 13046_2020_1557_MOESM1_ESM.pdf]
